# Bronchial Leech Infestation in a 15-Year-Old Female

**DOI:** 10.1155/2016/2372686

**Published:** 2016-09-26

**Authors:** Mohammad Ashkan Moslehi, Mohammad Hadi Imanieh, Ali Adib

**Affiliations:** ^1^Pediatric Pulmonology Division, Namazi Hospital, Shiraz University of Medical Sciences, Shiraz, Iran; ^2^Department of Pediatric Gastroenterology, Shiraz University of Medical Sciences, Shiraz, Iran; ^3^Student Research Committee, Shiraz University of Medical Sciences, Shiraz, Iran

## Abstract

Foreign body aspiration (FBA) is a common incidence in young children. Leeches are rarely reported as FBA at any age. This study describes a 15-year-old female who presented with hemoptysis, hematemesis, coughs, melena, and anemia seven months prior to admission. Chest X-ray showed a round hyperdensity in the right lower lobe. A chest computed tomography (CT) demonstrated an area of consolidation and surrounding ground glass opacities in the right lower lobe. Hematological investigations revealed anemia. Finally, bronchoscopy was performed and a 5 cm leech was found within the right B_7-8_ bronchus and removed by forceps and a Dormia basket.

## 1. Introduction

Foreign body aspiration (FBA) is a common life threatening event among children and mostly occurs between one and four years of age [1]. The clinical presentations can include persistent cough, dyspnea, choking, cyanosis, epistaxis, and hemoptysis [2].

Flexible bronchoscopy is the most sensitive and specific procedure for diagnosis of FBA [3]. Most FBAs are secondary to foods (seeds, nuts, beans, or fruit parts) or inorganic material including plastic and metallic objects [4]. However, leeches are rarely reported as FBA [5–7]. Leeches are blood sucking annelids. Leech infestation (hirudiniasis) can occur internally secondary to accidental ingestion from drinking from or swimming in contaminated water [8]. In this case report, we report a girl with bronchial hirudiniasis.

## 2. Case Presentation

A 15-year-old female was admitted to Namazi Hospital in Shiraz (southern Iran) in January 2015. She complained of occasional hemoptysis, hematemesis, coughing, vomiting, epistaxis, and melena approximately seven months prior to admission. She had a history of anemia that did not improve with ferrous sulfate supplementation. On physical examination, she was pale and afebrile. Fine crackles in the right lower lobe were present on pulmonary auscultation. Her abdomen was soft without organomegaly. She received octreotide and pantoprazole due to suspicion of gastrointestinal bleeding.

On admission, laboratory results revealed normocytic anemia (Table 1).

In stool exam, occult blood was 3+ and occult parasite was negative. Urine analysis showed normal values. Sputum examination for acid fast bacilli was negative.

**Figure 1:**
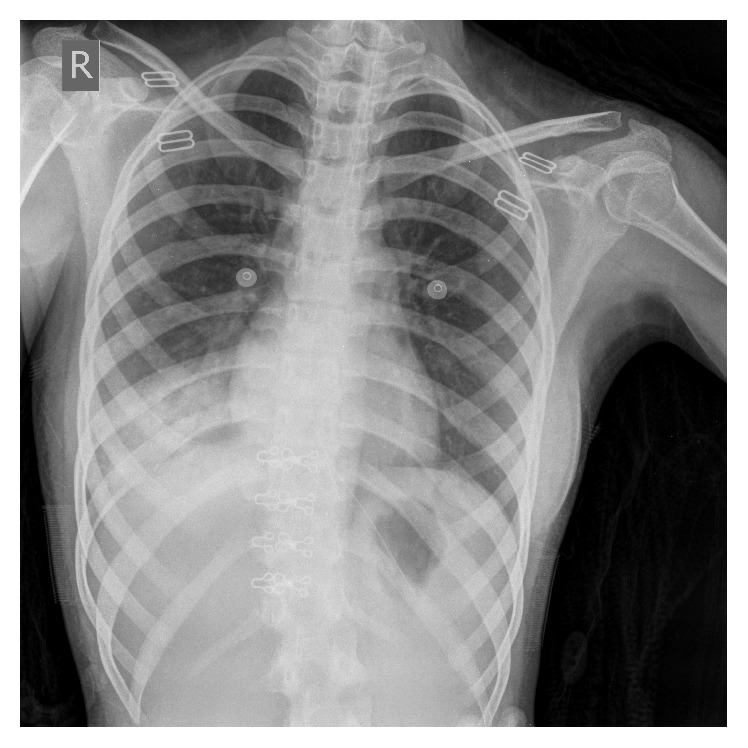
PA chest X-ray demonstrating right lower lobe opacity.

**Figure 2:**
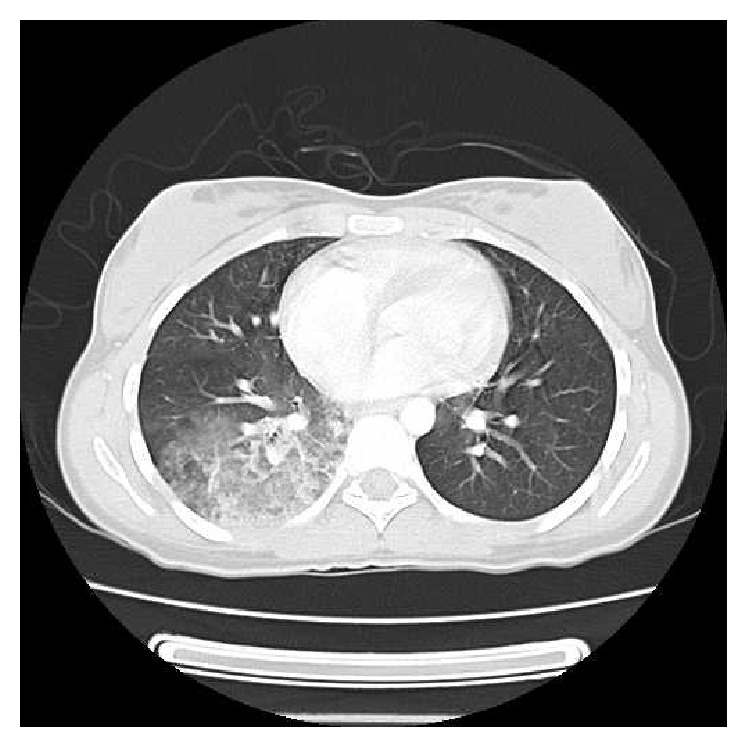
Spiral CT scan of the chest demonstrates an area of consolidation and surrounding ground glass opacity in the right lower lobe.

Chest X-ray showed a round hyperdensity in the right lower lobe (Figure 1). In abdomen and pelvic ultrasonography, the liver and spleen were normal in size and parenchyma. Gastrointestinal endoscopy revealed normal esophagus, stomach, and duodenum mucosa. Chest ultrasonography showed a minimal amount of free fluid in the right plural space. Spiral computed tomography (CT) scan with contrast of the chest and mediastinum was performed and demonstrated an area of consolidation and surrounding ground glass opacity in the right lower lobe (Figure 2). It also showed a filling defect in a segmental branch of the right bronchus suspicious for a clot, as well as bilateral reactive axillary lymph nodes.

**Figure 3:**
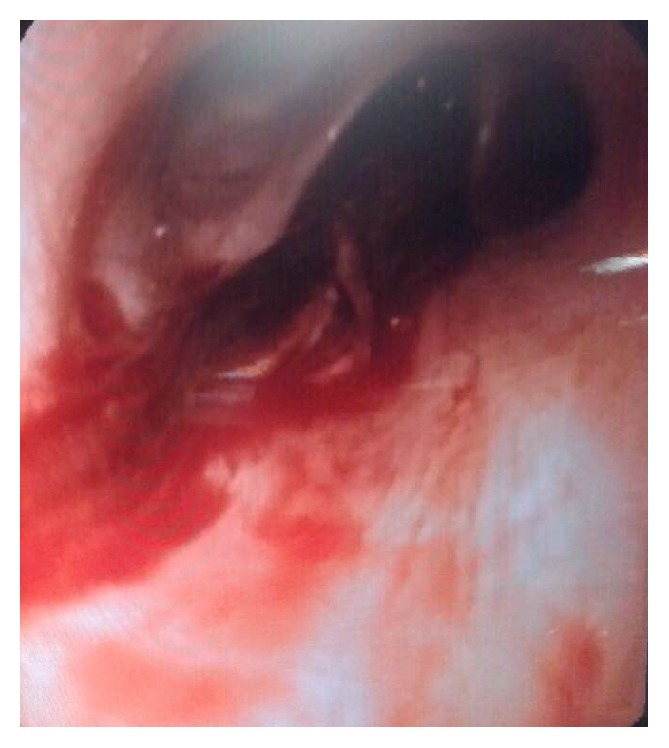
Image capture of the ruptured leech completely obstructing the right lower lobe bronchus (B_8_).

**Figure 4:**
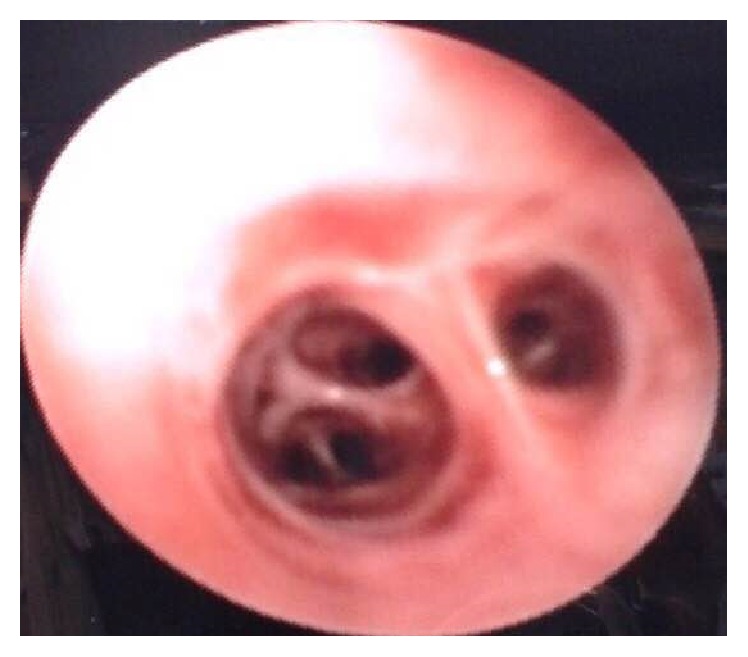
Right lower lobe bronchus (B_8_) following immediate removal of the leech.

**Figure 5:**
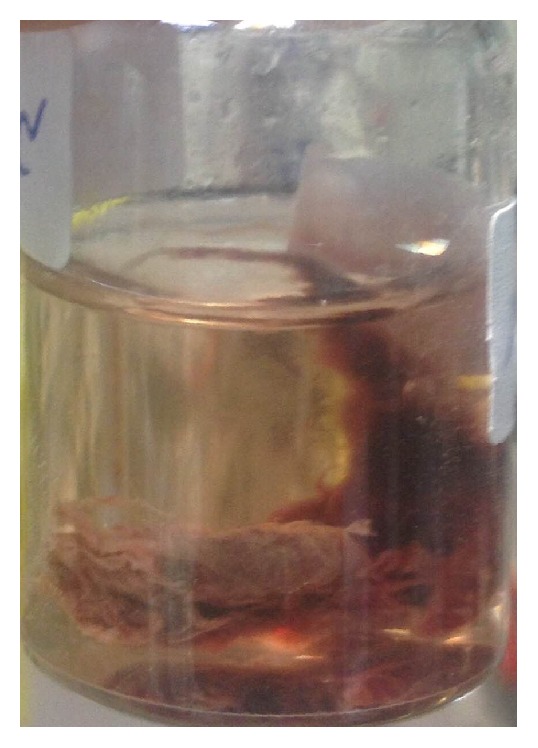
Leech after removal.

Because FBA was suspected, a flexible bronchoscopy under general anesthesia was performed. We sprayed lidocaine on the patient's vocal cords. A 5 cm worm-like undulating foreign body was found within the right lower lobe anteromedial bronchus (B_7-8_) (Figure 3). Also, there were some blood clots in the right mainstem bronchus (B_7_). We sprayed lidocaine on the worm to help detaching it. While trying to remove the worm by forceps, it ruptured and the leech's sucker remained attached to the mucosa. Next, the involved bronchus was washed with hypertonic saline (3%) solution to help removing the attached segment. A Dormia basket was passed through the remaining worm particle; it was then closed. After that, Dormia basket and the bronchoscope tube were removed.  Figure 4 shows B_8_ bronchus after complete removal of the leech. The animal's particle and the bronchoscopic tissue forceps biopsy were sent to the pathology lab, which confirmed that the foreign body was a leech.  Figure 5 shows the leech's body after removal.

## 3. Follow-Up

Repeat flexible bronchoscopy a month later revealed no leech, mass, or blood clot. Hematologic studies were repeated and her anemia was resolved (Table 1).

## 4. Discussion

Leeches are hermaphroditic parasites which live on blood. Leeches have been used for medical purposes for many centuries for a variety of conditions including dermatological diseases, reproductive system problems, inflammation, and venous congestion [9]. However, leeches can also cause disease such as internal hirudiniasis that may present with cough, hemoptysis, epistaxis, hematemesis, melena, dysphagia, hoarseness, and dyspnea [10]. The diagnosis is made sooner if the history includes drinking from infested waters, but in our case she reported to us that she drank from ab anbar (a traditional source of drinking water), after hirudiniasis was confirmed. Since our patient's clinical manifestation (hematemesis, vomiting, and melena) was similar to gastrointestinal bleeding, her diagnosis was delayed.

Several articles reported pharyngeal, laryngeal, and nasal hirudiniasis [6, 11, 12]. There are some rare reports of vaginal and ocular hirudiniasis [13, 14]. A similar case was a 40-year-old woman from China with tracheal leech infestation [7]. She had presented with hemoptysis, dyspnea, and a foreign body sensation in her throat, and she was diagnosed with asthma first [7]. In addition, a case report described a 7-year-old male from Ethiopia in whom the leech infestation was located in the proximal trachea [5].

Leeches secrete an anticoagulant agent called hirudin which makes the wound bleeding more than expected and it may persist even after the worm is removed. Therefore, as reported previously, hirudiniasis may lead to anemia such as in our case [15]. Treatment is removal of the leech as soon as possible to prevent complications.

Because leeches attach to the mucosa by strong suction, rupture of the worm is a risk. The detachment should be done carefully. The literature suggests lidocaine spray, hypertonic saline, or vinegar to assist with leech detachment [12, 16]. In our case, although lidocaine and hypertonic saline were instilled on the leech, it ruptured while attempting to detach it from the bronchial mucosa.

In conclusion, hirudiniasis should be considered in the differential diagnosis when a patient presents with hemoptysis, cough, dyspnea, and anemia. High suspicion is warranted when the patient is from a rural area or when they provide a history of drinking water from springs or rivers.

## Figures and Tables

**Table 1:** Laboratory results at admission and one month later.

Laboratory results	Admission	One-month follow-up
Hemoglobin (g/dL)	7.9	11.8
Red blood cell (/mm^3^)	2750000	3950000
Mean corpuscular volume (fl)	82.9	85
White blood cell (/mm^3^)	6900	5800
Neutrophils (%)	66.4	57
Lymphocytes (%)	28.1	37.9
Mixed (%)	5.5	5.1
Platelet (mm^3^)	241000	313000
Prothrombin time (sec)	14.5	12.7
International normalized ratio	1.21	1
Partial thromboplastin time (sec)	27	25

Erythrocyte sedimentation rate (mm/hr)	23	
Blood urea nitrogen (mg%)	13.5	
Creatinine (mg%)	0.6	
C-reactive protein (mg/dL)	3.9 (negative)	
